# Prevalence of hepatopathy in type 1 diabetic children

**DOI:** 10.1186/1471-2431-12-160

**Published:** 2012-10-06

**Authors:** Abdulrahman A Al-Hussaini, Nimer M Sulaiman, Musa D AlZahrani, Ahmed S Alenizi, Mannan Khan

**Affiliations:** 1 Division of Pediatric Gastroenterology, Hepatology & Nutrition, University of King Saud bin Abdulaziz for Health sciences Children's Hospital, PO box 59046, King Fahad Medical City, Riyadh, 11525, Kingdom of Saudi Arabia; 2 Division of pediatric Endocrinology Unit, Children Hospital, PO box 87341, King Saud, Medical City, Riyadh, 11642, Kingdom of Saudi Arabia; 3 Department of Radiology, Children's hospital, PO box 7855, King Saud, Medical City, Riyadh, 11117, Kingdom of Saudi Arabia

**Keywords:** Fatty liver, Hepatomegaly, Hepatic glycogenosis, Type 1 diabetes, Diabetes mellitus, Ultrasound

## Abstract

**Background:**

The Prevalence of liver disease among diabetics has been estimated to be between 17% and 100%. Most of these data were obtained from adult studies. The aim of our study was to screen for liver disease among type 1 diabetic children.

**Methods:**

Children with type 1 diabetes following in clinic have been examined for existence of liver disease, from November 2008 to November 2009. All were subjected to the following: History, physical examination, liver function tests, fasting lipid profile, HbA1C, and ultrasound of the liver. A hyperechogenic liver and/or hepatomegaly on ultrasound were attributed most likely to excess glycogen or fat in the liver, after negative extensive work-up to rule out other underlying liver disease.

**Results:**

106 children with type 1 diabetes were studied: age ranged between 8 months to 15.5 years, sixty two patients were females. Twenty two patients (21%) were identified to have abnormal findings on ultrasound of the liver: 10 patients had hepatomegaly and 12 had hyperechogenic liver. The group with hyperechogenic liver had poorer glycemic control than patients with normal liver (Mean HbA1c 12.14% Vs 10.7%; P value = 0.09). Hyperechogenic liver resolved in 60% at 6 months follow-up upon achieving better glycemic control.

**Conclusions:**

Hyperechogenic liver and/or hepatomegaly are not uncommon in children with type 1 diabetes and tend to be more prevalent among children with poor glycemic control. Type 1 diabetes related hepatopathy is reversible by optimizing glycemic control. Because of its safety, and reliability, ultrasound can be used to screen for hepatopathy in type 1 diabetic child.

## Background

Type 1 diabetes is a disorder of glucose metabolism that results from insulin deficiency secondary to autoimmune destruction of insulin-secreting β-cells. The prevalence of liver disease among diabetics is estimated to be between 17% and 100% [1-5]; fatty liver and hepatic glycogenosis being the predominant pathologies. Most of these data were obtained from studies of obese adults with non-insulin dependent diabetes. Since obesity may also be a major cause of hepatic abnormalities [1,4], it is difficult to identify diabetes as a specific factor for liver disease in obese diabetic adults. In a histopathological-based study in 91 adults with diabetes, fatty liver was diagnosed in 63% of patients with type 2 diabetes compared with 17% of patients with type 1 diabetes [2]. Because the frequency of obesity in adults with type 2 diabetes was higher than in patients with type 1 diabetes, it is not surprising that the incidence of fatty liver is different as well.

The pediatric literature about type 1 diabetes– related liver disease is largely limited to small case series or case reports for children presenting with symptomatic hepatic dysfunction during metabolic decompensation and ketosis [6-11]; hepatic glycogenosis being the most frequent etiology and much less frequent cases of hepatic steatosis. Screening metabolically stable children with type 1 diabetes for liver disease had been the subject of a single recent study [12] that showed a prevalence of abnormal hepatic ultrasound (hepatomegaly and/or hyperechogenicity) in 4.5% of screened children. However, ultrasound criteria used to define echogenicity and size of the liver were ill-defined. The aim of our cross-sectional and prospective study was to highlight the prevalence of diabetic hepatopathy in children with type 1 diabetes using well-defined ultrasound parameters to define echogenicity and size of the liver, in addition to clinical assessment and laboratory tests. Another objective was to evaluate the effect of implementation of measures to achieve glycemic control on diabetic hepatopathy.

## Methods

Children with type 1 diabetes in follow up in diabetes clinic in our tertiary care center were consecutively enrolled into the study. They presented initially and received therapy in our hospital and had not been treated for diabetes elsewhere. We excluded children with liver abnormality unrelated to diabetes or due to associated diseases. Children and their parents who have attended the diabetic clinic over the period from November 2008 to November 2009 and have accepted entry into the study were interviewed by the researchers and given an idea about the purpose of the study, and an informed consent form was signed. Children and their parents were asked to fill up a questionnaire to collect data about: demographics, duration of diabetes, insulin dosage, gastrointestinal or hepatic symptoms, and associated autoimmune diseases. Heights and weights were measured by clinic nurses using electronic scale and plotted on a sex and age appropriate growth chart, and body mass index (BMI) calculated. BMI ≥95^th^ centile for age and sex defined obesity and BMI ≥ 85^th^% - 95^th^% defined overweight. Hepatomegaly was assessed clinically by measuring the distance between the upper and lower borders of the liver at mid-clavicular line using a tape measure.

Blood was collected for: Liver enzymes (Alanine aminotransferase [ALT], aspartate aminotransferase [AST], alkaline phosphatase, gamma glutamyl transferase), total bilirubin, total protein and albumin, glycosylated hemoglobin (HbA1C), and fasting lipid profile. Hypercholesterolemia was defined as serum cholesterol > 5.15 mmol/L and hypertriglyceridemia was defined as serum triglyceride > 2.2 mmol/L. In our laboratory, normal ALT and AST are 30–65 IU/L and 15–37 IU/L, respectively. HbA1C > 10% defined poor glycemic control.

**Figure 1:**
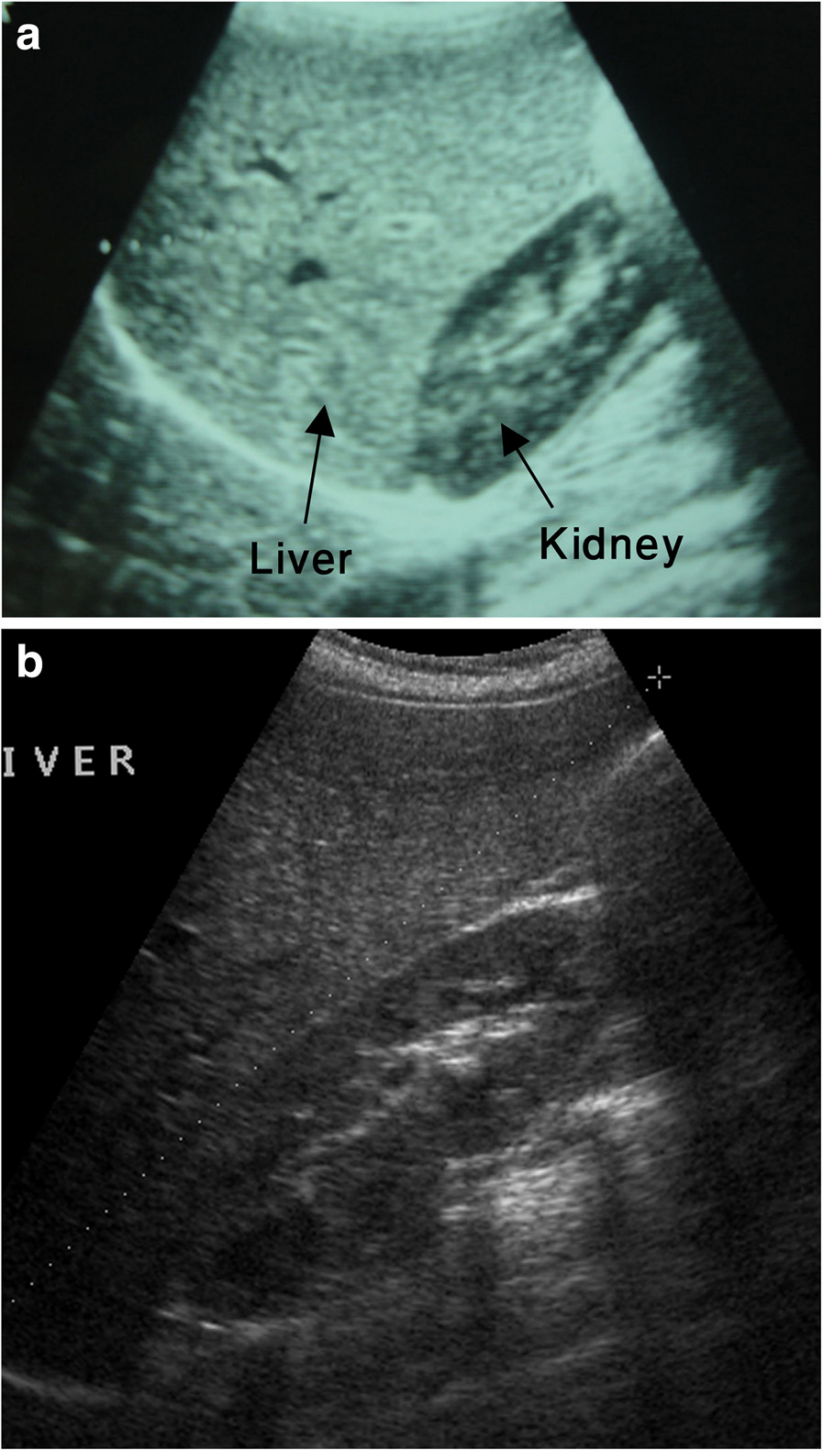
**(a) Marked hyperechogenicity of the liver resulting into large discrepancy between hepatic and renal echoes.** (**b**) Normal echogenicity of the liver after proper glycemic control.

Liver ultrasound was performed by a single senior pediatric radiologist to evaluate for presence of increased echogenicity or hepatomegaly. Ultrasound was performed using 3.5 –7.5 MHz curvilinear transducer on Aloka prosound SSD 4000 without any preparation. Radiologist was blind to clinical data of patient. Patients were placed on supine, right anterior oblique position to demonstrate the porta hepatis. Longitudinal images were obtained in the mid clavicular line. The length of the liver was measured from uppermost portion of the dome of diaphragm to the inferior tip. Uniform pattern of medium strength echoes from liver was considered as normal whereas increased strength of echoes resulting into marked liver-kidney echogenic discrepancy and poor definition of hepatic veins, portal vein, and crus of diaphragm was considered as increased echogenicity of liver [Figure 1(a)]. Length of liver above standard deviation (SD) of mean for normal liver size in children [13] was reported as enlarged liver. A liver size up to 2 cm above SD of mean for age and sex was considered as Mild Hepatomegaly, 2 to 4 cm above SD of mean defined moderate hepatomegaly, and liver size more than 4 cm above SD of mean defined severe hepatomegaly.

Presence of hepatomegaly and/or hyperechogenic liver on ultrasound abdomen was an indication for further laboratory work up to investigate other possible liver pathologies. The screening work up included: HBV surface antigen, Anti-HCV antibody, serum copper and ceruloplasmin, serum immunoglobulins, anti-nuclear antibody quantitation (by Immunoflourescence assay using Diasorin kit), smooth muscle antibody qualitative test (by Enzyme linked immunoassay using Diasorin kits), and Anti-Liver Kidney microsomal antibody (by Enzyme linked immunoassay using Diasorin kits), serum alpha 1 anti-trypsin, serum ferritin, serum iron, transferring saturation, and total iron binding capacity, urine for organic acids and plasma for amino acids, to evaluate for possibility of viral hepatitis B and C, wilson's disease, autoimmune liver disease, alpha 1 anti-trypsin deficiency disease, hemochromatosis, and metabolic disorder, respectively. We considered the finding of hyperechogenic liver and/or hepatomegaly on ultrasound and negative screening work up for underlying liver disease as consistent with a likely diagnosis of hepatic glycogenosis or less likely fatty liver. Liver biopsy was indicated if there is hepatomegaly and/or hyperechogenic liver on ultrasound and elevation of ALT and AST > 1.2 times of normal values. A liver biopsy was deemed unnecessary if there is hepatomegaly and/or hyperechogenic liver on ultrasound but normal liver function tests.

During the same study period, all 106 children with type 1 diabetes underwent screening for celiac disease using IgA tissue transglutaminase (IgA-TTG) [ELISA assay using Inova kit], endomyseal antibodies (EMA) [IFA assay using Inova kit], and total IgA level (unpublished data). Patients with positive IgA-TTG (> 20 units IU/L) and/or EMA underwent upper endoscopy and intestinal biopsies. Histopathology of the intestinal biopsies was graded according to Marsh classification [14].

Children with hyperechogenic liver and/or hepatomegaly and their parents were interviewed by an endocrinologist and dietitian and received counseling on importance of the liver finding and dietary intake was reviewed. In attempt to achieve glycemic control, parents and patient were encouraged to adhere to diabetic diet and, when appropriate, insulin dosage has been adjusted. At 3 months follow up, dietary compliance was reviewed by a dietitian. At 6 months follow up, blood was collected for HbA1C and ultrasound of liver was repeated. Radiologist was blind to the clinical data at repeated ultrasound.

The study has been approved by institutional review board and the procedures are in accordance with the Declaration of Helsinki.

### Statistical analysis

The data were analyzed using SPSS pc+ version 11.0 statistical software. Descriptive statistics (mean, standard deviation and proportions) were used to summarize the study variables. Student’s *t*-test for independent samples was used to compare the mean values of continuous study variables. The 95% confidence intervals for difference of mean were used. A p-value of less than 0.05 was used as a statistically significant.

## Results

Over the one-year study period, 106 children with type 1 diabetes were investigated: age ranged between 8 months and 15.5 years (Mean 8.5 years and standard deviation [SD] ± 2.8 years), sixty two were females and 44 males. Mean Age at diagnosis of type 1 diabetes was 6.3 ± 2.9 years (range 0.85 – 11 years). Mean time of duration of type 1 diabetes is 2.2 ± 2.1 years (range 0 – 8 years). Seven patients were newly diagnosed. Mean HbA1c was 10.7 ± 2.4%, and 61.3% of the patients had HbA1c more than 10%. Mean serum cholesterol was 4.16 ± 0.75 mmol/L (Normal 3.65 – 5.15 mmol/L) and mean serum triglyceride 1.02 ± 1.3 mmol/L (Normal 0 – 1.7 mmol/L). Only one child had hypercholesterolemia (serum cholesterol of 6.2 mmol/L) and another one had hypertriglyceridemia (serum triglyceride 3.5 mmol/L). None of the patients had elevated liver enzymes.

Ultrasound of the liver identified 2 groups of patients with abnormal liver imaging: a group of 11 children with hepatomegaly and normal echogenicity, One of them has β-thalasemia major so was excluded from the study, and another group of 12 children with hyperechogenic liver. None of the 22 patients had a history of hepatotoxic drug or family history of liver disease, and none had stigmata of chronic liver disease. The screening workup for possibility of underlying liver disease was negative. None of 106 children had gall bladder stones.

Table 1 shows the group of children with type 1 diabetes and ultrasound-diagnosed hepatomegaly. Clinical hepatomegaly was identified in 6 of the 10 patients; 3 cases of mild hepatomegaly and one case of moderate hepatomegaly on ultrasound were missed by clinical assessment. One patient was obese and none of the remaining was overweight. None of the group had short stature. None of the patients had elevated liver enzymes or hyperlipidemia. Mean HbA1c for this group was 9.4%. Comparison of the group of children with type 1 diabetes and hepatomegaly to patients with type 1 diabetes and no hepatomegaly shows no statistically significant differences as the age, duration of type 1 diabetes, HbA1c, insulin dose, and lipid profile are concerned.

**Table 1:** Children with type 1 diabetes and hepatomegaly

**Patient**	**Age (year)**	**Sex**	**Duration of diabetes (year)**	**BMI (Kg/m**^**2**^**)**	**U/S Liver size (cm) (severity)**
1	15	M	4	22.7	15 (mild)
2	3.5	F	0.5	18.5	10.4 (Mild)
3§	13	M	4	18.5	15.8 (Moderate)
4	8	M	1.5	16.57	12.6 (Mild)
5	4.5	M	3	16.6	10.5 (Mild)
6	9	F	0.4	13.7	16.7 (Severe)
7	11	M	3	17.5	12.2 (Mild)
8	4	M	3	15.7	10.2 (Mild)
9	4.5	M	1.5	16	11.8 (Moderate)
10	15.5	M	5.5	17.1	13.7 (Mild)

BMI: Body mass index, §: Obese patient, HbA1c: Glycosylated hemoglobin, U/S: Ultrasound, F: female, M: male.

Table 2 shows the group of patients with hyperechogenic liver on ultrasound. None of the group had elevated liver enzymes. Two patients were obese and none of the remaining was overweight. None of the group had short stature. Two patients had hyperlipidemia. Patient 2 had serum cholesterol of 6 mmol/L and patient 12 had serum triglyceride of 3.5 mmol/L. Comparison of the group of children with type 1 diabetes and hyperechogenic liver to patients with type 1 diabetes and no liver abnormality (Table 3) shows no statistically significant differences as the age, duration of diabetes, HbA1c, insulin dose, and lipid profile are concerned. Four patients (Patients 3, 5, 9, and 10) with hyperechogenic liver and type 1 diabetes (33%) were diagnosed to have celiac disease compared to eight patients with celiac disease in the non-hyperechogenic liver group (8.5%) (P value = 0.04).

**Table 2:** Children with type 1 diabetes and hyperechogenic liver

**Patient**	**Age (Year)**	**Sex**	**Duration of diabetes (Year)**	**BMI (kg/m**^2^**)**	**Hepatomegaly**
1	7	M	3	14.3	No
2*	10.5	M	4.5	17.9	No
3‡	12	F	3	15.5	No
4	9	F	2	16.89	Moderate
5‡	9.5	F	4.5	15.6	No
6	11.5	F	4.5	16	No
7§	8	M	7	22.6	No
8	3.5	M	0.1	16.9	No
9‡	11	F	0.3	17.7	No
10‡	13	F	6	23.6	Mild
11	11	M	0.3	14.8	No
12*§	10	M	0.5	35.1	Mild

BMI: Body mass index, HbA1c: Glycosylated hemoglobin, U/S: Ultrasound, *: patient has hyperlipidemia, §: Obese patient, ‡: Patient with celiac disease, F: female, M: male.

**Table 3:** Comparison of Mean values of study variables in relation to presence or absence of hyperechogenic liver in type 1 diabetes children

**Study variables (Mean)**	**Hyperechogenic liver Yes (12) No (84)**		**P - value**
Age (year)	9.7	8.3	0.11
Duration of diabetes (Year)	3	2	0.18
Height (Z score)	0.20	0.068	0.35
BMI (Z score)	0.66	― 0.12	0.14
HbA1c %	12.14	10.7	0.09
Insulin dose(Units/kg/day)	0.68	0.74	0.24
Triglyceride (mmol/L)	1.1	0.98	0.76
Cholesterol (mmol/L)	4.3	4.1	0.41

BMI: Body mass index, HbA1c: Glycosylated hemoglobin.

Table 4 shows data on ultrasound of the liver at 6 months follow up post- implementation of measures to achieve glycemic control in patients of both hyperechogenic liver and hepatomegaly groups. Six of 10 in the hepatomegaly group (60%) and 7 of 12 in hyperechogenic liver group (58%) had normal liver on ultrasound after achievement of better glycemic control [Figure 1 (b)]. Children with persistent hepatomegaly were non-adherent to diabetic diet and HbA1C worsened despite increasing the dose of insulin. All children who achieved normal liver size, except patient 5, achieved better glycemic control by good adherence to diabetic diet that led to subsequent reduction of insulin dose. Glycemic control in children with resolved hyperechogenic liver was achieved by good adherence to diabetic diet and increasing insulin dose. Five children had persisted hyperechogenic liver: patients 4, 6, and 12 were non-adherent to diabetic diet and patients 9 and 10, although their glycemic control improved, but they were not compliant on gluten free diet and had persistently high IgA TTG.

**Table 4:** Ultrasound of liver at 6 months follow up post-implementation of measures to achieve glycemic control in patients with hyperechogenic liver and/or hepatomegaly

**Hepatomegaly group**	**At baseline**		**At 6 months follow up**		
**Patient**	**Insulin (U/kg/d) HbA1C%**		**Insulin (U/kg/d) HbA1C% U/S liver**		
1	0.8	12	0.70	8.7	Normal
2*	0.5	10	0.75	11	Mild hepatomegaly
3	0.86	8.9	0.7	7.4	Normal
4*	0.59	7.4	0.5	8	Mild hepatomegaly
5	0.66	10.8	0.75	7.2	Normal
6	1	12.5	0.8	7.5	Normal
7*	0.62	8.1	0.7	8.8	Mild hepatomegaly
8*	0.88	8.9	0.8	9.5	Mild hepatomegaly
9	0.86	7	0.65	6.5	Normal
10	1.12	8.5	0.7	6.8	Normal
**Hyperechogenic liver group** ** Patient**					
1	0.73	11.6	0.85	8.8	Normal
2	0.48	13.6	0.7	8.2	Normal
3‡	0.69	10.6	0.8	7.5	Normal
4*	0.5	13	0.75	12.4	hyperechogenic liver
5‡	0.87	12.3	0.85	7.4	Normal
6*	0.6	14.5	0.9	13	hyperechogenic liver
7	0.55	12.7	0.75	9	Normal
8	0.64	9.1	0.70	8.2	Normal
9‡ ¶	0.46	14.1	0.75	10.5	hyperechogenic liver
10‡ ¶	0.75	13	0.90	9.8	hyperechogenic liver
11	0.72	13	0.80	8.8	Normal
12*	0.46	8.2	0.65	10.5	hyperechogenic liver

‡: patient with celiac disease; *: Non-adherent to diabetic diet; ¶: Non-adherent to gluten free diet.

## Discussion

The major finding of our study is the increased prevalence of of abnormal liver findings on ultrasound in children with type 1 diabetes (21%). The negative screening work up for underlying hepatic pathology (like viral hepatitis, autoimmune hepatitis, metabolic disease, Wilson’s disease, alpha 1 anti-trypsin deficiency, and hemochromatosis) makes the ultrasound finding of hyperechogenic liver and/or hepatomegaly in a child with type 1 diabetes likely due to excess glycogen or fat in the liver.

In type 1 diabetes, the insulin deficiency leads to low hepatic glucokinase levels resulting in decreased glucose uptake, and together with elevated glucagon levels stimulate glycogenolysis and gluconeogenesis [1]. Therefore, the low or absent insulin levels results in enhanced rate of glucose output by the liver and diminished hepatic glucose uptake. Treatment with insulin reverses these changes and normalizes uptake of glucose by hepatocytes and increases hepatic glycogen content. It has been shown in insulin-deficient rats given a single dose of insulin that glycogenosis persist for a substantial period after blood glucose returned to their initial elevated (insulin-deficient) values [15]. Thus the accumulation of hepatic glycogen is promoted by high cytoplasmic glucose concentration in presence of insulin. The Japanese literature reports 20 cases of diabetes mellitus-associated hepatomegaly and mild hepatic dysfunction attributed (especially in cases of type 1 diabetes) to chronic overinsulinization [16]. Hepatic glycogenosis has been reported to occur at first presentation of type 1 diabetes after receiving supraphysiological doses of insulin [11]. Therefore, longstanding hyperglycemia and overinsulinization are considered metabolic pre-requisites for hepatic glycogen storage. Also it has been suggested that patients who take excess insulin and treat the subsequent hypoglycemic episodes by administering glucose also promote hepatic glycogen accumulation. Thus a vicious cycle of hyperglycemia and intermittent insulin administration results in glycogenosis. The group of our patients with hepatomegaly had more significant number of hypoglycemic episodes and higher mean insulin dose compared to the group with no hepatomegaly. This might explain their liability to develop hepatic glycogenosis.

In type 1 diabetes, insulin deficiency stimulates lipolysis and increases the mobilization of peripheral free fatty acids, and their increased hepatic uptake enhances very low density lipoprotein and triglycerides synthesis [1]. The coexistent elevated glucagon levels inhibit hepatic triglycerides output. Therefore, hepatic steatosis in type 1 diabetes is thought to be a combination of an increased hepatic production of triglyceride and decreased removal. The group of our patients with hyperechogenic liver had poorer glycemic control, as indicated by their elevated mean HbA1C (12.14%), as compared with type 1 diabetes patients without hyperechogenic liver. This difference didn't reach statistical significance, which may be the result of relatively small sample size. Adequate insulin therapy can inhibit lipolysis and reverse the process. In contrast, in type 2 diabetes with high frequency of concurrent obesity, insulin insensitivity rather than the degree of glucose intolerance is predictive of the occurrence of hepatic steatosis [1]. As a result, weight loss has been shown to decrease the elevated hepatic free fatty acids concentration.

The pediatric literature about type 1 diabetes related liver disease is scant and mostly limited to small case series or case reports for children presenting with symptomatic hepatic dysfunction during time of metabolic decompensation and ketosis [6-11]; hepatic glycogenosis being the most frequent etiology and much less frequent cases of hepatic steatosis. Our study uncovers that type 1 diabetes related hepatopathy is not uncommon. Here, we report a prevalence of 21% (22 of 105) which is lower than the prevalence of fatty liver of 44% reported in adults with type 1 diabetes [17]. In the latter study, BMI was significantly higher in patients with type 1 diabetes and fatty liver as compared to those with type 1 diabetes without fatty liver, which suggests that obesity might be the major contributing factor for development of fatty liver in adults with type 1 diabetes. Unlike in adults with type 1 or type 2 diabetes and fatty liver, obesity was not a major factor in our patients; only 2 of 22 patients (9%) with type 1 diabetes related hepatopathy in our study were obese. This observation reinforces the thought that the pathogenesis for development of hepatopathy in type 1 diabetes and type 2 diabetes is different and subsequently the target of therapy will be different. Whereas adequate insulin dosage and adherence to diabetic diet are required to reverse hepatopathy in type 1 diabetes, weight loss helps improvement of insulin insensitivity in type 2 diabetes and ultimately inhibits lipolysis.

In a large study of 692 Egyptian children with type 1 diabetes, El-Karaksy et al. [12] reported a prevalence of 4.5% of abnormal liver findings (hyperechogenicity and hepatomegaly) on ultrasound as compared to 21% in our study. The higher frequency of liver abnormality on ultrasound in our study could be attributed to the poorer glycemic control of our patients; 61.3% have HbA1c > 10% as compared to 24% in El-Karaksy's study. Another explanation for variation in prevalence of abnormal liver findings between the two studies could be related to the methodology used. In El-Karaksy's study, ultrasound criteria used to define echogenicity of the liver were not mentioned, definition of hepatomegaly was not stated, and radiologist was not blind to clinical data. In our study, we have considered several reliable ultrasound parameters to define echogenicity of the liver and we defined hepatomegaly in reference to normal liver size in age and sex matched children [13]. In addition, the ultrasound was performed by a single radiologist blind to clinical data to minimize bias. In a recent meta-analysis on the diagnostic accuracy and reliability of ultrasound for the detection of fatty liver, the use of different ultrasound criteria to define echogenicity of liver was among the most important causes for the variation of estimates of fatty liver between studies [18]. In the same met-analysis, the accuracy of ultrasonographic parameters of fatty liver definition were evaluated individually and the sensitivities of liver to kidney contrast and vessel wall brightness, two criteria we used to evaluate for fatty liver in our study, were 98% and 81% respectively, with specificity between 93% to 95% for both parameters.

Data from our study indicate that type 1 diabetes related hepatopathy can be silent unless screened for by ultrasound. Majority of the cases reported in the pediatric literature were for children presenting in metabolically decompensated conditions and in diabetic ketoacidosis states; these cases manifested hepatic symptoms and evidence of liver dysfunction. The liver enlargement in these cases may be so fast that it causes stretching of the liver capsule and abdominal pain. The design of our study to screen metabolically stable children may have contributed to the asymptomatic nature of hepatopathy in our cohort.

Our data suggest that the prognosis of type 1 diabetes related hepatopathy is excellent and the process is reversible with optimal control of blood glucose. Diabetic hepatic glycogenosis is a benign condition that tends to resolve in response to establishment of glycemic control [6,19] and no report exists of progression to fibrosis. However simple steatosis may incite an inflammatory response leading to steatohepatitis. The natural history of steatohepatitis is still being defined, however progression to fibrosis occurred in 48% of patients [20] and cirrhosis had been reported in 10 – 15% [20,21]. The increased prevalence of fatty liver among patients with type 1 diabetes has important implications on their clinical care. Detection of hepatic steatosis at an early stage in children with type 1 diabetes might offer them the advantage of reversibility by proper glycemic control. Persistence of hyperechogenic liver in patient 9 and 10 despite better glycemic control could be due to celiac disease, a recognized cause of fatty liver [22].

Ultrasound of liver is a valuable non-invasive tool to diagnose liver disease in children with type 1 diabetes. All of the 22 children with abnormal liver findings on ultrasound were asymptomatic, and demonstrated normal liver enzymes; therefore the dependence on clinical data and liver function tests to detect liver disease could have missed those cases. Ultrasound has a sensitivity of 80% and a specificity of almost 100% for steatosis [18,23], especially for the detection of moderate to severe fatty infiltration. The sensitivity and specificity of ultrasound for detection of fatty liver is similar to that of imaging techniques (computed tomography or magnetic resonance imaging) [18]. Therefore, we think that ultrasound of liver is indicated to screen for hepatopathy in diabetic children particularly those with persistent poor glycemic control. However, histopathological demonstration of glycogen or fat accumulation in hepatocytes remains the gold standard to diagnose glycogenosis or fatty liver and to differentiate simple steatosis from steatohepatitis. The ultrasound finding of hepatomegaly is nonspecific and, in a child with type 1 diabetes, could be due to increased accumulation of fat or glycogen in hepatocytes. Our inability to confirm the ultrasound finding of fatty liver or hepatic glycogenosis on histopathological ground and lack of control group are limitations in our study. None of our patients showed an elevation of liver transaminases; a criterion we have set in our study protocol to justify doing liver biopsy.

## Conclusion

Type 1 diabetes related hepatopathy is not uncommon and tends to be more prevalent among children with poor glycemic control. Type 1 diabetes related hepatopathy is reversible by optimizing glycemic control. Because of its low cost, safety, reliability, and accessibility, ultrasound can be used to screen for hepatopathy in children with type 1 diabetes.

## Abbreviations

ALT: Alanine aminotransferase; AST: Aspartate aminoTransferase; BMI: Body Mass Index; HbA1C: Glycosylated hemoglobin.

## Competing interest

Authors disclose no conflict of interest and have no financial relationships to disclose. We acknowledge that the sponsor of this research “King Abdulaziz City for Science and Technology” (KACST) thru a grant No. LPG-10-41, had no role in the study design, data collection or analysis, manuscript writing, or submission for publication.

## Authors’ contributions

All of the following authors have participated sufficiently in the work to take full responsibility for the content: A. Al-Hussaini has contributed to conception of idea and design of the study, data research and design; analysis and interpretation of data; writing the manuscript. NS has researched data, analysis and interpretation of data and reviewed and edited manuscript. MA has researched data, analysis and interpretation of data and reviewed and edited manuscript. A. Alenazi has researched data, contributed to discussion and edited the manuscript.MK has performed ultrasound and contributed to discussion. All authors read and approved the final manuscript.

## Pre-publication history

The pre-publication history for this paper can be accessed here:

http://www.biomedcentral.com/1471-2431/12/160/prepub
